# Role of the ventral portion of intermediate arcopallium in stability of female Bengalese finch song preferences

**DOI:** 10.3389/fpsyg.2024.1490858

**Published:** 2025-01-14

**Authors:** Austin Coulter, Jonathan F. Prather

**Affiliations:** Program in Neuroscience, Department of Zoology and Physiology, University of Wyoming, Laramie, WY, United States

**Keywords:** mesopallium, nidopallium, pallium, ventral tegmental area, song evaluation, electrolytic lesion, songbirds, decision making

## Abstract

The process of decision making is a complex procedure influenced by both external and internal conditions. Songbirds provide an excellent model to investigate the neural mechanisms of decision making, because females rely on acoustic signals called songs as important stimuli in directing their mate choice. Previous experiments by our group and others have implicated secondary auditory brain sites in female evaluation of song quality, including the caudal portions of the nidopallium (NC) and mesopallium (CM). Recent pathway tracing experiments reveal a convergence of those sites onto a third region, the ventral portion of the intermediate arcopallium (AIV), suggesting that AIV may also play an important role in song evaluation and mate choice. Here we combined behavioral testing with lesion inactivation to investigate the role of AIV in song preference of female Bengalese finches (*Lonchura striata domestica*). Inactivation of AIV was associated with destabilization of rank ordering of song preferences. These data suggest a model in which the convergence of auditory activity in AIV plays an important role in female perception of song quality and production of courtship behaviors. Together with previous results that also demonstrate a role for the auditory areas that converge onto AIV, these findings extend the experimental tractability of this emerging animal model of sensory perception and decision making.

## Introduction

Decision making enables organisms to navigate physical and social worlds. This process involves evaluation of both internal volitional conditions and external stimuli. Cases in which an action is initiated in response to external stimuli are referred to as sensory-based decision making. The processes of sensory-based decision making enable animals to detect signals, perceive their characteristics, and take action based on the information present in that sensory signal. Avoiding predation through scanning for visual or auditory cues, finding quality food by scent or coloration, and interpreting advertised qualities of potential partners before engaging in mating are a few examples of important decisions that an individual must make. Decisions that lead an individual to higher fitness are adaptive, whereas those that lead to lower fitness are maladaptive, and the success of an individual depends on its ability to make adaptive decisions. A deeper understanding of the neural basis of decision making and a mechanistic approach to facilitating adaptive decisions requires a better understanding of the cells and neural circuits that underlie this cognitive process.

Songbirds are an excellent animal model to study the neural basis of decision making. In many species of songbirds such as the Bengalese finches (*Lonchura striata domestica*) studied here, males perform ornate learned vocalizations called songs, but females cannot sing. Instead, females evaluate the quality of male songs and other traits and use that information to select their mate (Catchpole and Slater, 2008). Mates provide many benefits in nest defense, food procurement, and partnership in raising the clutch, so it is adaptive for females to choose a mate that provides them with advantageous benefits (Long et al., 2022). That decision is affected by many factors such as plumage and courtship behaviors, but among these influences, song is such an important signal that females will solicit copulation in response to song even if no male is physically present (Clayton and Prove, 1989; Dunning et al., 2014; MacDougall-Shackleton et al., 1998). Thus, songs provide a unimodal stimulus that plays an essential role in a fundamentally important sensory-based decision making process.

Previous studies of song preferences in females have led researchers to investigate how song preferences and the associated actions are encoded in the brain. Individual females are generally consistent in their preferences across time and tests, indicating that preferences are a consistent trait of adult individuals (Dunning et al., 2014). Importantly, different females disagree about which male they find most attractive, suggesting that there are important individual-specific factors that shape song preferences (Dunning et al., 2014). Consistent with that idea, preferences are associated with sensory and social experience during early life and in some cases throughout adulthood (Anderson et al., 2014; Sewall et al., 2018). This experience-dependent aspect reveals that song preferences and the associated expression of courtship behaviors are at least partially learned behaviors, leading researchers to question how they are represented in activity of cells in the central nervous system.

Specific sites in the female songbird brain have been implicated in song evaluation and mate choice (Brenowitz, 1991; MacDougall-Shackleton et al., 1998; Sewall et al., 2018; Tomaszycki and Blaine, 2014). These sites include secondary auditory cortical areas, the caudal mesopallium (CM), and the caudal nidopallium (NC) (MacDougall-Shackleton et al., 1998; Tomaszycki and Blaine, 2014; Van Ruijssevelt et al., 2018; Woolley and Doupe, 2008). CM has been implicated in perception and evaluation of individual songs. This is evident in greater activity in response to attractive songs, specifically with respect to songs that are behaviorally directed toward a specific female, versus undirected songs, which are broadcast to no specific receiver (Diez et al., 2019; Elie et al., 2019; Van Ruijssevelt et al., 2018; Woolley and Doupe, 2008). Findings from studies in which CM was inactivated through lesions have supported its role in song evaluation. Inactivation of CM is associated with an alteration of female preference for male songs, specifically, females become less selective following lesioning of CM (Lawley et al., 2022; MacDougall-Shackleton et al., 1998).

Activity in NC has also been associated with song evaluation, and its role is especially evident regarding familiarity and memory of specific songs. Neurons in NC are more active when a female hears a song with which she is very familiar, such as her father’s song, and when she hears a song that is completely novel (Terpstra et al., 2006; Van Ruijssevelt et al., 2018; Woolley and Doupe, 2008). Activity in the representation of very familiar songs could arise from the female remembering her past experiences with the song, while distinct activity associated with unfamiliar songs could come from the process of recording the song to memory (Terpstra et al., 2006; Van Ruijssevelt et al., 2018; Woolley and Doupe, 2008). Similar to what was found with inactivation of CM, inactivation of NC also induces changes in a female bird’s song preferences. This inactivation does not affect the female’s ability to hear or to recognize individual males based on their songs, but it does cause females to become less selective for songs that she previously found distinctly attractive (Lawley et al., 2022; Tomaszycki and Blaine, 2014). Together, these data reveal important roles for NC and CM in female song evaluation and mate choice. The central roles that CM and NC play in this animal model of sensory-based decision making lead to the additional questions of how pathways in the brain provide auditory input to those areas, and how additional networks link those areas to downstream sites to shape the associated behavioral responses.

**Figure 1:**
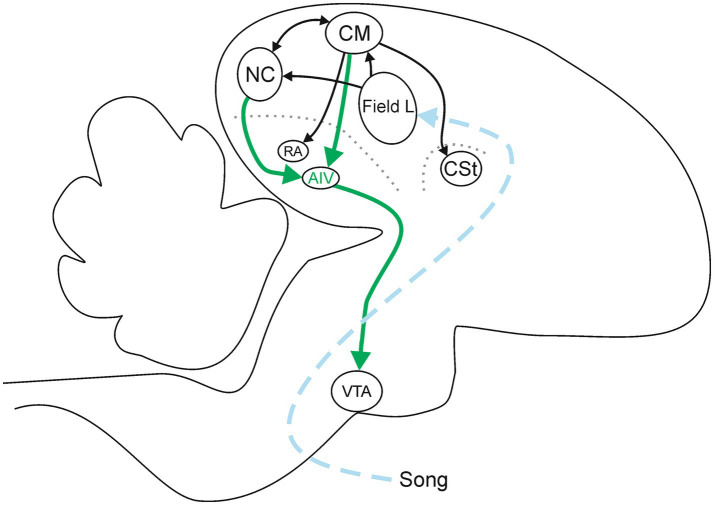
Sagittal depiction of some of the pathways relevant to AIV. The ascending dashed arrows summarizes the auditory afferent pathways (blue). The thick solid arrows are projections from afferent sites CM and NC onto AIV, and from AIV onto its downstream target in VTA (green). Thin solid arrows indicate pathways interconnecting other elements of this network (black). The dotted lines represent laminae that are used as anatomical landmarks to help identify RA, AIV, and CSt (gray). Diagram represents a summary of published data (Dunning et al., 2018; Mandelblat-Cerf et al., 2014; reviewed in Prather, 2013; Reinke and Wild, 1998; Vates et al., 1996).

CM and NC receive input directly from the primary auditory cortex (Field L, Figure 1) (reviewed in Prather, 2013). At the level of CM and NC, these sites are interconnected, providing a local path through which each can modulate activity in the other site, and through which complex computations involving auditory stimuli could emerge, such as song quality and identity (Bloomston et al., 2022; Dunning et al., 2018; Vates et al., 1996). In addition to these local connections, CM also projects to additional targets through which it could influence behavioral responses. These include input to a motor pallial area implicated in control of respiration and vocalization (robust nucleus of the arcopallium, RA), projections into the caudal striatum (CSt), and input to a pallial area implicated in learning and imitation in males but largely unexplored in females (the ventral portion of the intermediate arcopallium, AIV) (Dunning et al., 2018; Mandelblat-Cerf et al., 2014; Reinke and Wild, 1998; Vates et al., 1996) (Figure 1). Additional investigations of the projections that emerge from NC have revealed that NC projects sparsely to CM, and NC also sends robust projections into AIV (Bloomston et al., 2022; Mandelblat-Cerf et al., 2014; Vates et al., 1996) (Figure 1). Thus, there is a convergence from two auditory areas that are implicated in perception and evaluation (CM and NC) onto a single target site (AIV). This architecture suggests that AIV could play an important role in the processing of auditory information emanating from CM and NC.

AIV is well positioned to participate in song evaluation and formation of preferences. AIV projects monosynaptically to brain sites that have been implicated in behavioral motivation and reward. Specifically, AIV projects to neurons in the dopaminergic ventral tegmental area (VTA) (Chen et al., 2019; Kearney et al., 2019; Mandelblat-Cerf et al., 2014). Studies linking behavioral testing and pharmacological experimentation have revealed that dopamine (DA) serves a similarly rewarding role in songbirds as it does in mammals (Schroeder and Riters, 2006). These dopaminergic neurons in VTA project back to pallial areas in songbirds, providing a circuit through which sensory-evoked activity in CM and NC could affect dopamine levels and thereby affect perception and behavioral motivation (Gale et al., 2008). At the hub of this circuit lies AIV, suggesting that it could also play a key role in female song evaluation and mate choice.

The present study tested the hypothesis that inactivating AIV results in behavioral changes in the female Bengalese finch model of song-based decision making. We employed well-established means of measuring an individual female’s song preferences, and we tested the degree to which those preferences changed following bilateral lesions in AIV. If no changes emerged, this would implicate pathways other than the convergence of CM and NC onto AIV as key contributors in song evaluation. Alternatively, any change in song preferences would implicate AIV as an important component in song perception and mate choice. Here we investigate the role of AIV and consider the possible pathways through which it may exert its influence in this emerging model of decision making.

## Materials and methods

### Overview of experimental design

We tested our hypothesis using well-established methods of identifying a female’s degree of preference for representative songs performed by individual males of her own species (Dunning et al., 2014; Dunning et al., 2020; Elie et al., 2019). In that approach, female Bengalese finches typically produce many calls in response to some songs and few or no calls in response to others (Dunning et al., 2014; Dunning et al., 2020; Elie et al., 2019). This easily detectable and quantifiable behavioral response is closely correlated with the expression of copulation solicitation displays, enabling researchers to use the number of calls produced in response to each song as a means of identifying each female’s mate preference (Dunning et al., 2014). With those preferences identified for each female studied here, we electrolytically lesioned AIV bilaterally. After recovery, each female’s song preferences were measured again and analyzed for any behavioral change. At the end of behavioral testing, neural tissue was processed and analyzed to confirm the placement of lesions and the bird’s sex, as evident in clear sexual dimorphism of song nuclei such as HVC (Nottebohm and Arnold, 1976).

### Care and housing of experimental subjects

Two commercial breeders (Louie’s Aviary, Cranston, RI; New York Bird Supply, Bronx, NY) sourced the Bengalese Finches which were kept in social cages (41 × 31 × 24 cm) on a 16:8 h light:dark cycle with food and water available *ad libitum*. We identified male birds by the presence of song in audio recordings, while we identified females by the absence of song in continuous recordings over a four-day period. Only adult females (>120 days post-hatch) were used for experimentation. Between phases, females lived in female-only cages (41 × 31 × 24 cm) to limit exposure to male song, as this tends to facilitate responsiveness in behavioral assessment of song preference (Dunning et al., 2014; Dunning et al., 2020; Elie et al., 2019; Lawley et al., 2022).

### Preparation and presentation of song stimuli

We collected songs from six adult male conspecifics and one adult male heterospecific (zebra finch, *Taeniopygia guttata*) to create the stimuli used for preference assessment. All these songs were novel to the female subjects at the start of this study. We sampled songs from males who were housed individually and recorded continuously for 4 days, and then randomly selected five songs as the stimuli associated with that male. As an individual male’s song performances differ from each other, we compiled a set of five songs into a stimulus set for each male, with each song in the sets separated by 1.2 s of silence to allow females to be exposed one stimulus from each male that contained a representative set of song performances (total duration of each stimulus set ranged from 42 to 52 s). Ten seconds of silence separated each set of stimuli, and we randomized the sequence of song presentation in each behavioral test. Each set of song stimuli corresponded with an individual male identity. Stimuli were played through a speaker located in the behavioral testing chamber (5–10 cm from the edge of the cage, distance varied as the female moved within the cage; 70 dB of stimulus intensity as measured at the center of the cage). A behavioral trial consisted of one presentation of each set of stimuli from each male, and multiple behavioral trials were performed in each phase of testing.

### Behavioral testing to identify song preferences

Behavioral testing consisted of two phases. Phase 1 occurred before surgery to identify each female’s individual preferences. Phase 2 occurred after surgery to investigate the degree to which each female’s preference had been altered due to lesion placement or sham surgery (control birds were also tested in a second phase with no surgical intervention in between). Song preferences were determined by a female’s responsiveness to male song, calling more in response to songs they found more attractive. The methods employed to measure song preference were identical in each phase and followed established procedures to measure song-based mate preference in this species (Dunning et al., 2014; Dunning et al., 2020; Elie et al., 2019; Lawley et al., 2022). First, we placed a female into a sound attenuation chamber and gave her at least 30 min to acclimate to the new environment (Dunning et al., 2014). Each attenuation chamber contained a housing cage (41 × 24 × 21 cm), a speaker, and a camera. This arrangement enabled experimenters to remotely monitor the female’s behavioral and vocal reactions (e.g., calls) to song stimulus sets played through a speaker.

To ensure that preferences were assessed using an adequate dataset, we enforced the criterion that a female must call at least 10 times in response to one song or four times to two or more songs (following the procedures of Dunning et al., 2014). Valid trials that met this criterion were included in the analysis. We measured each female’s preference over four valid trials in each phase of testing. We excluded females that were unresponsive in Phase 1 and did not achieve four valid trials within a maximum total of eight trials from further testing and returned them to the colony (*n* = 6 birds). Testing in Phase 2 was identical to tests in Phase 1, except no birds were excluded because of behavioral features in Phase 2. If birds but did not pass four out of a maximum of either trials in Phase 2, the four trials where they were the most responsive were used (*n* = 3 birds in total: 2 experimental birds, 1 sham bird). This was done to ensure that we did not bias the dataset to exclude the possibility that lesions could cause decreased responsiveness. Each phase of testing consisted of four trials in which the seven stimulus sets were presented in a randomized sequence. Only two behavioral trials were performed for each bird within any given day, to minimize the possibility of response habituation in the females.

We measured a female’s degree of preference for different males by counting the number of calls that she produced during playback of each set of stimuli from each male (Dunning et al., 2014; Dunning et al., 2020; Elie et al., 2019). The number of calls that a female produced in response to each set of songs from each male were then expressed as a percentage of the total calls recorded in that trial, and we compared those percentages to quantify the female’s preference. For each female, this approach revealed one song that evoked the greatest number of calls (the “most-preferred” song), at least one song that evoked the smallest number of calls (the “least-preferred” song), and a range of intermediately-preferred song stimuli (Dunning et al., 2014; Dunning et al., 2020; Elie et al., 2019).

### Treatment groups

We divided birds into control (*n* = 9), sham (*n* = 7), and experimental (*n* = 12) groups. Each group was investigated in two phases of behavioral testing. For experimental birds, Phase 1 consisted of four trials followed by a bilateral surgical placement of lesions in AIV. Afterwards, we monitored the birds closely, recognizing full recovery as vigorous expression of eating, drinking, calling, and performance of other typical behaviors such as grooming. After at least 24 h of recovery, we measured each female’s song preference again in Phase 2 consisting of another four trials of behavioral testing. For sham birds, the surgical procedure was performed between Phases 1 and 2, including insertion of the lesioning electrode, but no lesion was placed. For control birds, tests of preference were performed before and after a period of time corresponding to the typical period of surgical recovery, but no neural manipulation was made between Phases 1 and 2.

### Surgical placement of lesions

After Phase 1, we bilaterally lesioned AIV in females of the experimental group (n = 12 birds). We anesthetized the female with 3% isoflurane in pure oxygen, then positioned her head in a stereotaxic rig with the beak secured at a head angle of 45 ° below horizontal. Anesthesia was maintained at a lower level of isoflurane (typically 1–2%) throughout the surgery. We made bilateral craniotomies as small windows in the inner leaflet of the skull at 0.25 mm anterior and 2.2 mm lateral from the posterior bifurcation of mid-sagittal sinus (“y-zero”), targeting AIV in both the left and right hemispheres. We then lowered a small carbon fiber electrode wrapped in borosilicate glass (Carbostar-1, Kation Scientific), 3.1 mm deep into the cranial tissue. Using the electrode, we passed 60 μA through the 20 μm exposed tip in intervals of no more than 15 s, with at least equal durations of rest between each period of stimulation. All lesions were between 900 and 6,000 μA × sec (average lesion of 3,473 ± 1,176 μA × sec; variable in each bird according to ability to pass current at each lesion site). With the lesion placed, we withdrew the electrode, closed the craniotomies, and covered the craniotomies with a biologically inert silicon epoxy (Kwik-Sil, World Precision Instruments, FL). Then we rejoined skin and sealed the incision with medical adhesive (Vetbond, 3 M), and applied a local anesthetic to minimize discomfort (2.5% lidocaine and 2.5% prilocaine cream, Hi-Tech Pharmacal, NY). With the surgery complete, we removed the bird from the rig and moved it to a recovery cage where it was housed individually and monitored closely throughout recovery (typically 1 h). We recognized initial recovery by the bird standing upright, hopping, drinking water, and defecating. We monitored the birds for at least an additional 24 h prior to any behavioral testing to ensure complete recovery. All birds recovered well, were included in subsequent behavioral testing, and remained healthy throughout observation. After the period of post-operative recovery, each female entered Phase 2 of behavioral testing. This surgical process was the same for the sham group, except no current was passed and no lesion was placed. After the end of Phase 2 of testing, we sacrificed the birds and collected their neural tissue for histological processing.

### Locations of electrolytic lesions

The goal in this study was to inactivate neurons in the portion of intermediate arcopallium (AI) that receives synaptic input from either CM and/or NC. Previous experiments have revealed that the ventral portion of that area (AIV) receives monosynaptic input from CM and NC, and neurons in that region send monosynaptic projections to dopaminergic brainstem area VTA (Mandelblat-Cerf et al., 2014). Partial lesions of AIV are sufficient to have a behavioral impact in male birds (Mandelblat-Cerf et al., 2014), and other groups have also shown that partial lesions of other sites can have significant impacts on behavior (Cardin et al., 2005; Del Negro et al., 1998; Lawley et al., 2022; MacDougall-Shackleton et al., 1998). Here we sought to inactivate AIV through partial or complete lesions. As there is not complete agreement in the literature regarding the boundaries of AIV, sites we affected included the region of the arcopallium that receives synaptic input from CM, NC, or both (Mandelblat-Cerf et al., 2014; Mello et al., 2019). This area included tissue immediately surrounding RA and immediately anterior of RA, as well as tissue between those sites and the inferior surface of the arcopallium and extending 200 microns laterally of edge of RA. This region is based on the definitions of AIV described by Mandelblat-Cerf et al. (2014) and is consistent with definitions used by Mello et al. (2019), which provides more detailed anatomical description of anatomical boundaries. All our lesions affected that area, and none of our lesions impacted any tissue beyond the dorsal arcopallial lamina (LAD).

### Histological processing to confirm lesion locations

After the end of Phase 2 of behavioral testing, we used an overdose of isoflurane to euthanize each bird then perfused them transcardially with cold physiological saline (0.9%) followed by 4% paraformaldehyde in phosphate buffer (PFA). We removed the brain and placed it in 4% PFA for 24 h or until it was completely saturated, as determined by the tissue sinking to the bottom of the solution when gently agitated. Once saturated, we moved the brain to a solution of 4% PFA in 30% sucrose to cryoprotect the tissue. It remained there until it was saturated, as determined by sinking to the bottom of the solution. We then froze the cryoprotected brain, parasagittaly sliced it into 50-micron sections, and mounted it on gelatin-coated microscope slides. After drying overnight, the tissue was rehydrated using descending alcohol concentrations, stained with cresyl violet, dehydrated with ascending alcohol concentrations, and then cleared with xylene. This process marks the endoplasmic reticulum of the cells, making the individual cells distinct and enabling useful anatomical landmarks to be resolved. We examined the tissue under a microscope (BX51 Fluorescence Microscope, Olympus) to ensure lesion accuracy and to confirm that all subjects were female birds.

### Statistical analysis

To investigate the possible impact of lesions on each birds’ ability to call, we compared the total number of calls between Phase 1 and Phase 2 for each treatment group using Student’s t-test. To measure possible changes in the selectivity of song preference resulting from AIV lesions, the selectivity of preference (difference in the percentage of calls per trial) was compared between Phase 1 and Phase 2 for each group using an analysis of variance (ANOVA). We also investigated possible changes in song preference rankings for each bird between Phase 1 and Phase 2. The number of calls in response to each stimulus were used to rank the song stimulus sets from one to seven, with the ranking of one given to the stimulus with the most calls, and the ranking of seven given to the stimulus with the fewest calls. If two stimuli received the same number of calls, they were given the same rank, rounding to the higher rank (i.e., if two song stimuli were tied for the lowest number of call responses, each one would be ranked number 6). The ranks were then plotted at the group level, with the rank in Phase 1 plotted along the x-axis, and the rank in Phase 2 plotted along the y-axis. Next, a Pearson’s correlation coefficient was calculated to quantify comparison of the relationship between the rank ordering in each phase. The three treatment groups (experimental, sham, and control groups) were then compared with an analysis of covariance (ANCOVA), and significance was determined with Tukey’s honestly significant difference (HSD) test.

### Ethical note

All procedures were approved by the University of Wyoming Animal Care and Use Committee, and all procedures were in compliance with recommendations from that group and state and federal regulations governing the housing of songbirds.

## Results

### AIV lesioned females expressed behavioral indicators of song preference

**Figure 2:**
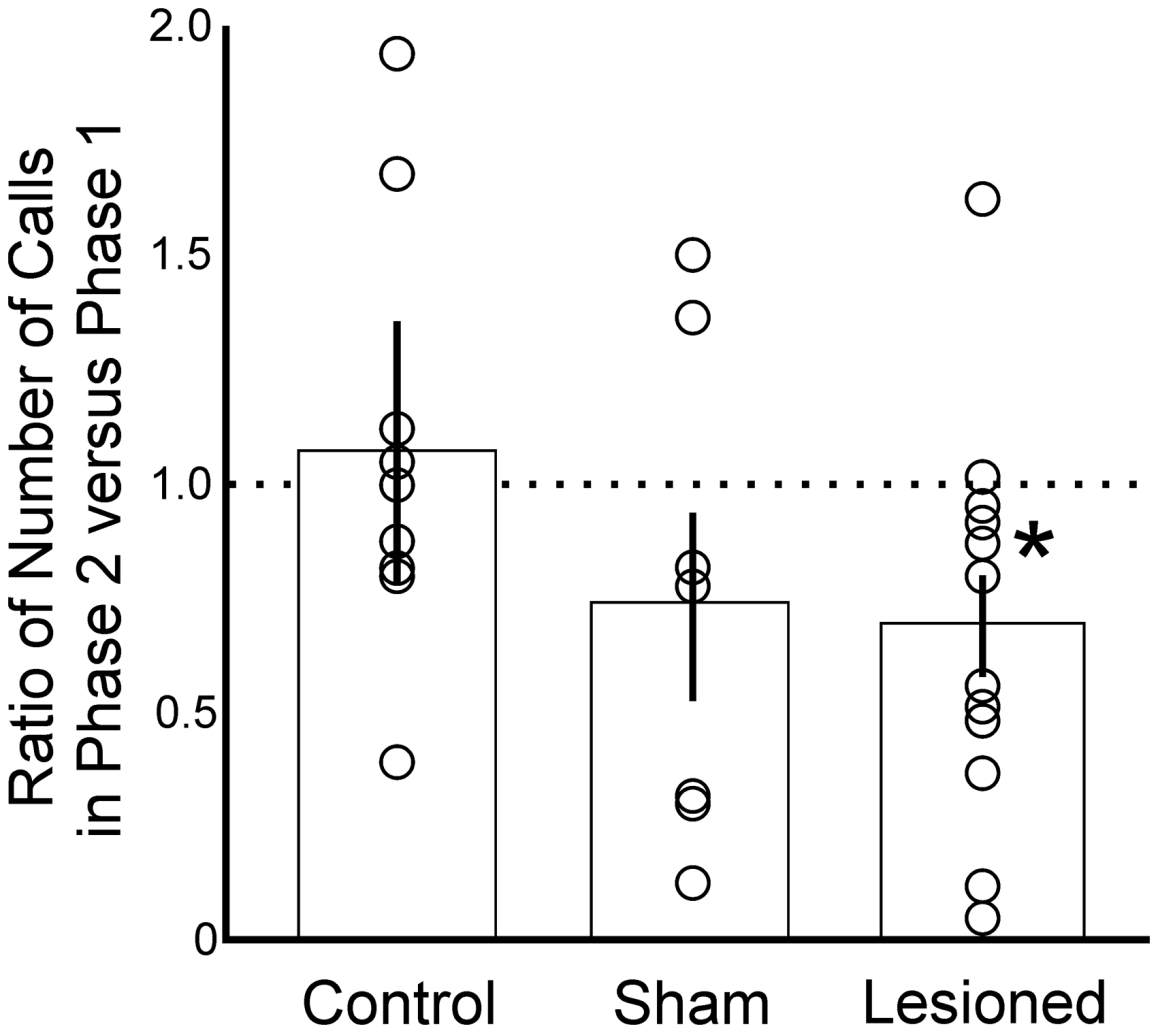
Experimental birds produced fewer calls following a lesion to AIV (Phase 2), but still expressed sufficiently robust courtship behaviors (calls) to enable detection of song preferences. This was evident as a decrease in the number of calls among females in the lesioned group (*p* < 0.03, *n* = 12) but not among birds in the control (*p* = 0.45, *n* = 9) or sham (*p* = 0.11, *n* = 7) groups. Open circles indicate individual data points for each condition. Columns and error bars indicate mean ± SE for each condition. * indicates significance, *p* < 0.05.

We investigated the degree to which lesions may have induced changes in females’ willingness or ability to perform behavioral indicators of song preference. We compared the total number of calls that each female performed in response to all seven sets of song stimuli across all four trials before versus after receiving a lesion in AIV (Figure 2). Following the lesion (Phase 2), experimental females expressed significantly fewer calls (*p* = 0.03, Student’s *t*-test, *n* = 12). On average, birds in the experimental group expressed 68% of their original call output, revealing a reduction in total output but confirming that females were still capable of expressing robust behavioral output to reveal their preferences (Figure 2). Birds in the sham group also expressed fewer calls (74%), although this reduction was not significant (*p* = 0.11, Student’s *t*-test, *n* = 7). This decrease is consistent with previous reports that surgical manipulations can cause reduced responsiveness of songbirds in subsequent behavioral tests (Yamahachi et al., 2020). No such reduction was observed for birds in the control group (*p* = 0.45, Student’s *t*-test, *n* = 9) (Figure 2). The control birds on average increased their output by 7% in Phase 2, but this effect was dominated by one bird that increased its behavioral output two-fold from Phase 1 to Phase 2. Without that bird, the group expressed an average of 96% of its original output. Together these data reveal that birds in the experimental group remained capable of robustly expressing behavioral indicators of their song preference, but the total magnitude of their response decreased after the lesion.

### AIV lesions induced changes in individual female song preferences

**Figure 3:**
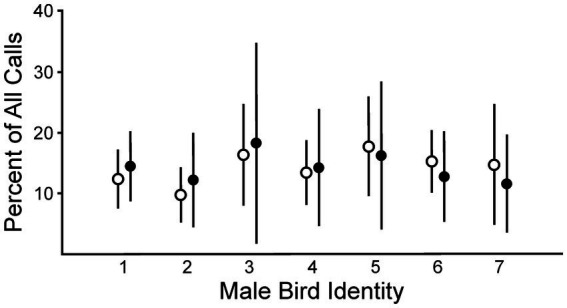
There were not significant differences between groups before (Phase 1, open circles) versus after lesions in AIV (Phase 2, closed circles) in females’ responses to all males considered together (ANOVA, *p* = 0.99, df = 1). In contrast, there were differences in females’ evaluation of the quality of different males (ANOVA, *p* < 0.001, df = 6). Data are shown as means ± SD (*n* = 12 birds).

We compared the number of calls that each female produced in response to songs from each male before versus after lesions placed in AIV. Responses to each song stimulus set were expressed as a percentage of the total calls detected within each of the four trials and then averaged across the four trials to determine the responsiveness for each phase. This enabled us to make comparisons across individuals and phases of testing. No changes were detected in the population-level response to male songs from Phase 1 to Phase 2 (Figure 3; ANOVA, *p* = 0.99, df = 1). There were also no differences among treatment groups in their population-level responses (ANOVA, *p* = 0.99, df = 2). These data indicate that there were no systematic changes in the females’ responses across the array of male song stimuli. As has been previously reported, males differed in their relative degree of attractiveness across female subjects (ANOVA, *p* < 0.001, df = 6). These data revealed that there were differences in the perceived attractiveness of the songs from each male. There were also differences in each female’s ranking of those males, as different females identified different males as their most attractive song stimulus set (Dunning et al., 2014; Dunning et al., 2020; Elie et al., 2019; Lawley et al., 2022). Thus, individual-specific differences in song preference were evident in this measurement of a continuous behavioral variable. In additional analyses, we incorporated these individual-specific differences, and effects of lesions became apparent.

We analyzed possible changes in the degree to which an individual male’s songs became more or less attractive to each female as a result of lesions in AIV. For example, the song that a female found the most attractive in Phase 1 could become systematically less attractive following lesioning, and/or songs that a female identified as less attractive could become systematically more attractive in Phase 2. To test for this possibility, we computed each female’s ranking of the seven song stimuli in the behavioral tests of Phase 1. To ensure that we had sufficient resolution to reliably identify each female’s ranked preferences, we enforced the criterion that each female must express at least 100 calls in both Phase 1 and Phase 2. Twenty of our 24 birds passed this criterion, with 8/9 birds retained in the control group, 6/7 birds retained in the sham group, and 10/12 birds retained in the experimental group. The significance of previous results did not change when these birds were also excluded from previous analyses.

**Figure 4:**
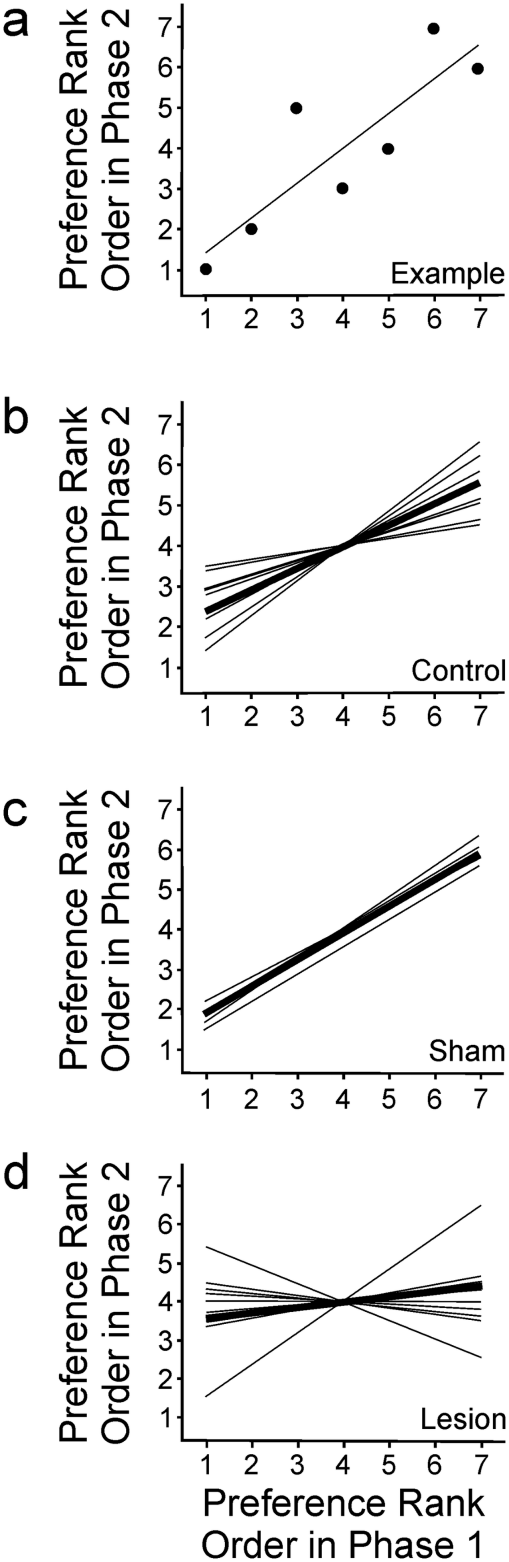
To compare the rankings of song preference in Phase 1 and Phase 2, ranks in Phase 1 were plotted along the x-axis, and the same bird’s rankings of the same songs in phase 2 were plotted along the *y*-axis. **(A)** This example depicts the rankings for one bird in the control condition. Individual points are the rankings for each song stimulus, and the line depicts a linear regression for those data. In the following plots, average slopes for individual birds are shown as thin lines in each group, and average slope of all data within each group is indicated by the thick line in each panel. **(B–D)** Rank ordering of song preference was unchanged from Phase 1 (*x*-axis) to Phase 2 (*y*-axis) in **(B)** control birds (*n* = 8) and **(C)** sham surgery birds (*n* = 6). In contrast, rank ordering was significantly disordered as a result of lesions to AIV in **(D)** experimental birds (*n* = 10).

Song rankings of individual females in Phase 1 and Phase 2 were compared using plots like those show in Figure 4. In such depictions, completely stable rank ordering of song preference after lesioning would be evident as linearly arranged data with a slope of one. In contrast, if preferences were completely inverted, the data would still be linearly arranged but would have a slope of negative one. Data ranging between these extremes and diverging from either of those expectations would indicate a change in the female’s rank ordering of preference following lesion placement. We do not assert that cognition in female birds regarding their preference for one song or another necessarily involves a strict linear ranking, but this measure is especially useful in measuring and comparing song preferences in different conditions.

Data from birds in the control group revealed a correlation between females’ rankings in Phase 1 and Phase 2 (Pearson correlation, *R*^2^ = 0.28, *p* < 0.001). This general stability of preference was also evident in sham birds, as they also yielded correlated data (Pearson correlation, *R*^2^ = 0.48, *p* < 0.001). It is not clear why there was greater correlation of responses in Phase 1 and Phase 2 in the sham group than in the control group, although it may have been influenced by the sample size in the sham group. The general stability of song preference in these groups indicates that preference was not significantly altered by the surgical intervention. In contrast to the stability observed in control and sham groups, the data from experimental birds varied much more widely, evident as a lack of significant correlation between data in Phases 1 and 2 (Pearson correlation, *R*^2^ = 0.07, *p* = 0.26).

Experimentally induced changes in rank ordering were also reflected in the linear characterizations of the rank ordering patterns in each dataset (Figures 4A–D). Control birds revealed a linear regression (*p* < 0.001) with slope of 0.53 (Figures 4A,B). The slope being less than one could be explained by the subtle differences in preference from one test to another, which have been described previously for females of this species (Dunning et al., 2014). Consistent with that pattern, these data also revealed that control birds expressed some variability within an overall pattern of general stability of preference. Similarly, birds in the sham group also revealed a linear regression (*p* < 0.001) with slope of 0.67, again indicating general stability of rank order preference (Figure 4C). In contrast, birds that received lesions in AIV revealed data that were variable between Phase 1 and Phase 2 to such a degree that they were not consistent with a linear regression (*p* = 0.26) with a slope of 0.14 (Figure 4D). That variability in the lesioned group is especially evident in the case of one bird for which preferences were largely unchanged (line moves up as it moves to the right) and another bird for which preferences were starkly changed (line moves down as it moves to the right) (Figure 4D). A comparison of the slopes estimated for each of the conditions (ANCOVA, *F* = 5.48, df = 2, *p* < 0.01) revealed no difference between the control and sham groups (Tukey’s HSD, *p* > 0.05), but each of those was different from the highly variable responses observed in the experimental condition (Tukey’s HSD, *p* < 0.05 in both cases). Together with the low correlation for data observed for rank ordering of song preference in experimental birds, these data reveal that females’ song preferences were significantly destabilized following lesioning of neural tissue in and around AIV.

### Lesion locations and sizes

**Figure 5:**
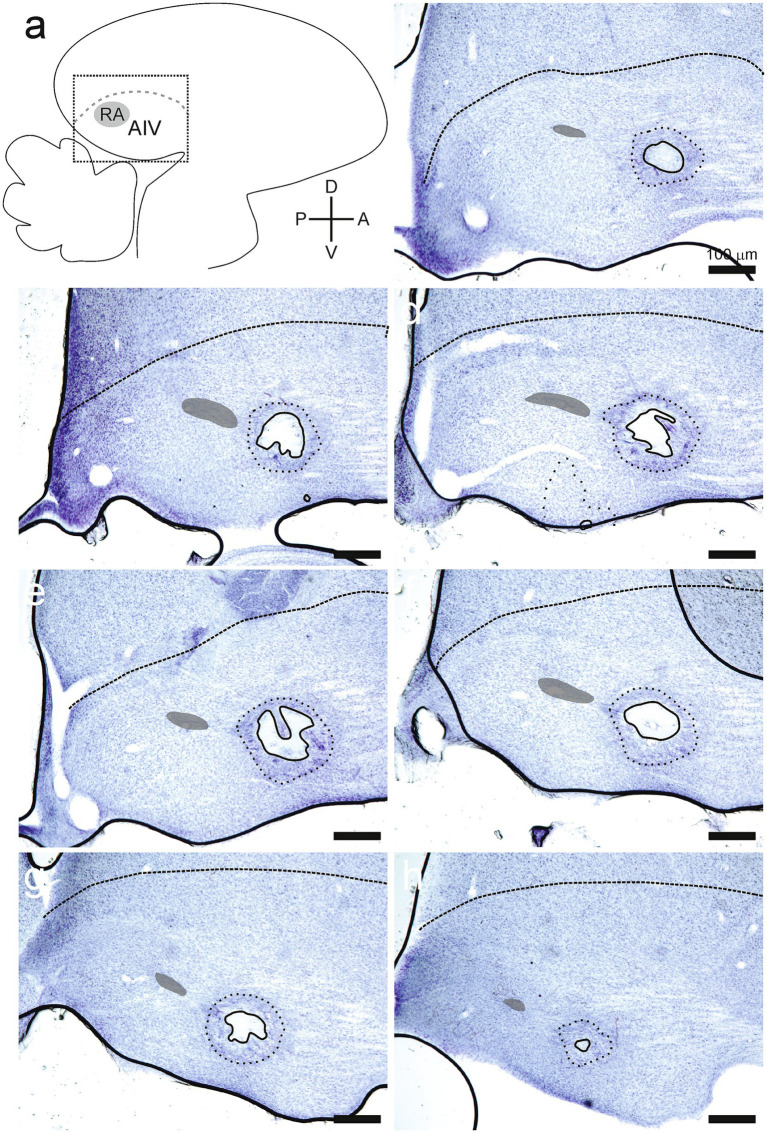
Lesions were placed into the ventral portion of the intermediate arcopallium (AIV). **(A)** All data are shown in parasagittal sections. The dotted square indicates the region shown in subsequent panels. **(B–H)** All these panels are taken from serial 50-micron sections in the right hemisphere of the same bird, with sections proceeding from most lateral **(B)** to most medial **(H)**. The lesion was not evident in the sections immediately bracketing these serial sections. These data are representative of the extent of lesion effects within the arcopallium. Lesions were identified by an absence of cells (lesions, solid lines). Partially affected regions surrounding each lesion were recognized by discolored tissue and fewer somas (coronas, dotted lines). Dashed lines in the upper portion of each section indicate the lamina that defines the superior margin of the arcopallium (LAD). Nucleus RA is indicated by a partially opaque area within the arcopallium of each section.

**Figure 6:**
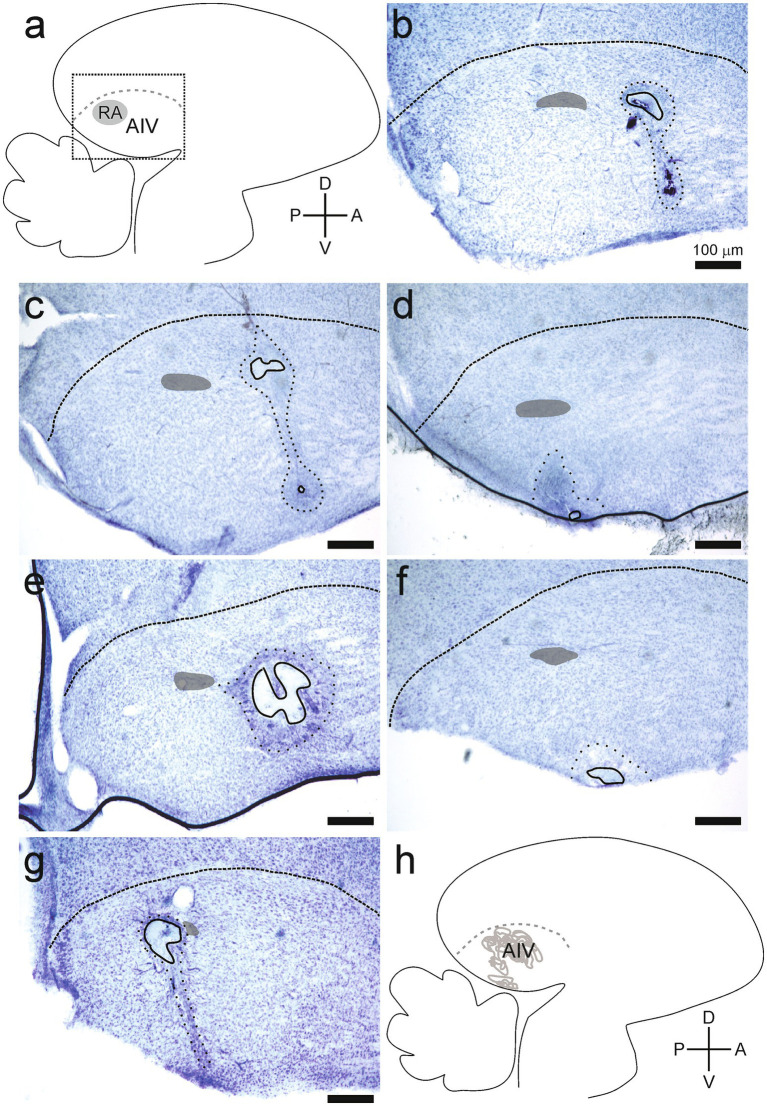
Lesions affected the ventral portion of the intermediate arcopallium (AIV). **(A)** Anatomical data are shown in parasagittal sections, and all tissue is presented as in Figure 5. **(B–G)** Lesions and the surrounding coronas were identified as described in Figure 5. Each panel is taken from a different bird. None of the lesions included in this study affected tissues beyond the LAD (*n* = 24 hemispheres, 12 birds). **(H)** Locations of all lesions are summarized as overlaid outlines in one panel (*n* = 24 hemispheres).

Lesions were identified as sites where experimental manipulation caused elimination of somas in that site (Figures 5, 6). The extent of lesioned tissue in each direction was determined for each of the 24 hemispheres in the experimental birds. On average, the lesion extended 233 ± 104 microns in the medial-lateral direction, 268 ± 205 microns in the dorsal-ventral direction, and 277 ± 126 microns along the anterior–posterior direction (all values are mean ± SD; Figures 5, 6). Every lesion spanned at least a portion of AIV, as defined using previously described parameters (Mandelblat-Cerf et al., 2014; Mello et al., 2019). Each lesion was surrounded by an area where the tissue was discolored, and cell numbers were decreased but not eliminated. We referred to these surrounding areas as the corona associated with each lesion. On average, the corona extended 417 ± 103 microns in the medial-lateral direction, 765 ± 335 microns in the dorsal-ventral direction, and 534 ± 188 microns in the anterior–posterior direction (all values are mean ± SD; Figures 5, 6). Three hemispheres from two birds did not have a detectable lesion but did have a visible corona. For these hemispheres the lesion size was declared to be 50 microns (one section of tissue in histological examination), as tissue was clearly damaged; but the extent of the lesion was small enough to not appear in either of two adjacent 50 micron tissue sections.

Lesions also affected areas surrounding AIV, including the RA (*n* = 9/24 hemispheres), tissue immediately medial to the extent of AIV (*n* = 5/24 hemispheres), tissue immediately lateral to the extent of AIV (*n* = 3/24 hemispheres), and tissue immediately dorsal of the extent of AIV (*n* = 9/24 hemispheres). The extent of these additional effects was typically small, extending 63 ± 106 microns medially or 25 ± 77 microns laterally. The extension of dorsal damage was determined by its proximity to LAD as there are no markers to unmistakably differentiate the Dorsal Arcopallium (AD) and AIV. For each type of impact beyond the extent of AIV (including RA, extending medially, extending laterally, or extending dorsally), no differences in any of the parameters described in these Results were detected. Specifically, there were no differences in the numbers of calls that the bird produced in Phase 1 versus Phase 2 (Student’s *t*-test, RA, *p* = 0.93; medial, *p* = 0.38; lateral, *p* = 0.98, dorsal: *p* = 0.81). Tests of rank ordering of song preference also revealed no differences between birds in which the lesion did or did not extend beyond the margins of AIV (ANCOVA, df = 1, RA: *p* = 0.23, medial: *p* = 0.82, lateral: *p* = 0.37, dorsal: *p* = 0.14). Therefore, we made no distinctions among these subsets of lesions that all impacted AIV but extended additional distances in various directions. We interpreted the results from our experimental group as indicating changes in song preference that emerged as a result of inactivating cells in AIV.

## Discussion

We electrolytically lesioned portions of AIV to investigate the role of activity in that area affecting female song preference and mate choice. Lesions caused a significant decrease in the total number of calls that female birds produced in response to male songs, but sufficient calls remained to enable us to resolve female preferences both before and after lesioning. As previously reported, females differed in their evaluation of the attractiveness of different songs (Dunning et al., 2014). Investigation of those individual-specific differences revealed that lesions in AIV caused females to change their expression of song evaluation and mate preference. This was evident in the changes in rank ordering of song preference such that a female’s degree of preference for the songs produced by each of many males was destabilized. This change was due to inactivation of AIV, as preferences became significantly less stable in lesioned birds, but control birds or birds that received sham surgeries remained stable in their preferences. Together, these results reveal that a loss of activity in the cells and pathways that course through AIV is associated with alteration but not a complete destabilization of preference for individual males and their songs.

### Functional contributions of pallial areas in the evaluation pathway

These results implicate AIV as a key element in the pathway through which auditory signals are processed and used to influence the production of female courtship behaviors. Although areas outside of AIV were affected by some lesions, AIV was affected in every lesion, and none of our results revealed differences in behavioral impact for any lesions that affected the surrounding tissue. Therefore, the observed behavioral changes are associated with changes in AIV rather than areas immediately adjacent to AIV. The impact of the electrical lesions on AIV included damage to somas in that site as well as fibers of passage that course through AIV. Our results are not able to disambiguate the contributions of local somas versus fibers of passage, but our observation that the pattern of behavioral changes was indistinguishable for lesions that affected a variety of locations within AIV suggests that activity within that area played an important role rather than specific pathways that may have been affected by some lesions but not others. An important goal of future research will be to further resolve the contributions of specific pathways to specific aspects of song preference and mate choice. In the remainder of this text, we consider possible circuits and cellular mechanisms through which AIV and other portions of the evaluation pathway may exert their influence on mate choice.

One pathway that may have been impacted by the lesions placed in this study was the projections from CM to RA. RA has been implicated in affecting the timing of call production, but lesions in RA do not prevent females from producing calls (Benichov et al., 2016; Simpson and Vicario, 1990). This role may account for calls being generally preserved but fewer in number in lesioned birds. Alternatively, decreased production of otherwise unchanged calls may have been a residual effect of the surgical manipulation (Yamahachi et al., 2020), as calls were decreased in sham birds although not to a significant degree, which may have been influenced by a smaller sample size. These results suggest that even though RA was affected in some of the lesions in this study, effects on the pathway from CM to RA are unlikely to have played a major role in the changes in song preference observed here.

**Figure 7:**
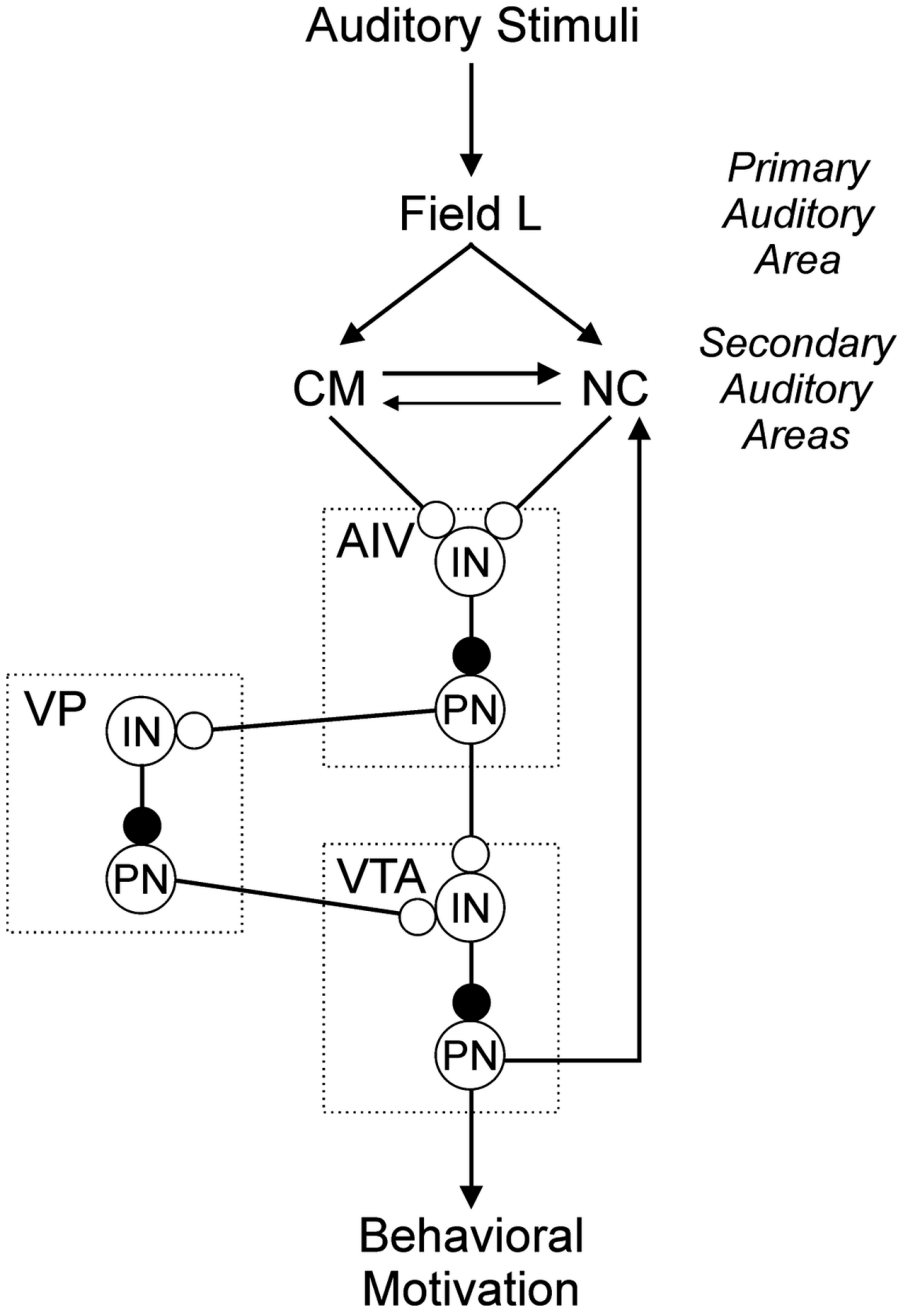
AIV is a central component in a network through which songs are evaluated and perception is used to shape the production of courtship behaviors. Auditory stimuli arrive in the primary auditory cortex (Field L), and output is passed to the secondary auditory pallial areas (CM and NC). Those secondary areas converge onto AIV, and output is passed to the ventral pallidum (VP) and dopaminergic cells in VTA. VP also projects to dopaminiergic cells in VTA (references for circuit connectivity are as described in the legend of Figure 1). Here we posit a detailed composition of this circuit in which interneurons (IN) and projection neurons (PN) may contribute to that processing and may account for observed patterns of neural activity in these sites. Lines indicate axons. Open circles indicate excitatory connections and filled circles indicate inhibitory connections.

The primary neural pathway through which lesions in AIV could influence preference and decision making is likely the connection between the auditory centers CM and NC and the dopaminergic center VTA (Figure 7). CM activity is greater in female songbirds when they hear songs that they find more attractive (Elie et al., 2019; Van Ruijssevelt et al., 2018; Woolley and Doupe, 2008). Electrophysiological recordings of individual neurons in CM reveal that those neurons are selective for very specific song features, with the activity of different cells associated with different song features (Chen and Meliza, 2018). This precise selectivity may enable the populations of CM neurons to represent the occurrence of specific songs, and that information may contribute to assigning value to a specific song and thus shaping mate choice for the associated singer.

The auditory area NC is also likely to play an important role in the perception of song quality and the decision of mate choice. Activity in NC has been associated with song preference in females, as females become more active when a female bird hears a song she finds attractive (Canopoli et al., 2014; Gentner et al., 2001; Lawley et al., 2022; Menardy et al., 2012; Terpstra et al., 2006; Tomaszycki and Blaine, 2014; Van Ruijssevelt et al., 2018; Woolley and Doupe, 2008). Deactivation of neurons in NC leads to a decrease in song selectivity (Lawley et al., 2022; Tomaszycki and Blaine, 2014). This role in shaping selectivity and preference for specific songs and the associated singers likely emerges from NC’s role in auditory memory, as NC has been implicated in song recognition and learning in both males and females.

Through the reciprocal interconnection of CM and NC, it seems likely that both participate to varying degrees in the comparison of a song stimulus to previous experience and the evaluation of the quality of that stimulus (Bloomston et al., 2022; Dunning et al., 2018; Vates et al., 1996). This connection unites the specific evaluation-related features of CM with the memory-related functions of NC, providing a possible network through which auditory signals are evaluated in service of decision making.

As we progress through the evaluation pathway beyond CM and NC, the next important pallial site is AIV. The anatomical convergence of CM and NC onto AIV suggests that there could be a functional convergence as well. Ultimately, any impact of the computation that may occur in AIV would be dependent on the projections from AIV to its downstream targets. An important downstream recipient of AIV’s projections is VTA (Mandelblat-Cerf et al., 2014). Neurons in VTA are dopaminergic, and dopamine (DA) is an essential neurotransmitter in systems that underlie behavioral motivation and reward (Gale et al., 2008; Kearney et al., 2019) (reviewed in Riters, 2012). It is intuitive to suspect that DA could play a key role in song preference and mate choice, as preferences are subjective evaluations that are associated with rewarding outcomes, and quality is judged by certain stimuli being more rewarding than other stimuli (reviewed in Ikemoto and Panksepp, 1999; Wise, 1980).

A final pathway that could also contribute to the emergence of preferences and mate choice is the interconnection between AIV and the ventral pallidum (VP) in the forebrain. The function of VP remains unclear, but it appears to play a role in song perception and learning. Specifically, VP receives input from portions of the anterior forebrain pathway (AFP), which is implicated in the song learning process of male songbirds (Benichov et al., 2016; Clayton, 1988; Gale and Perkel, 2010; Nottebohm et al., 1976; Simpson and Vicario, 1990; reviewed in Prather, 2013). VP is also connected to VTA, providing an additional connection through which perception could influence behavioral motivation and reward (Chen et al., 2021; Chen et al., 2019; Gale et al., 2008; Kearney et al., 2019). The data of this study highlight the importance of an area (AIV) that also projects to VP. This pathway between AIV and VP could also be a link through which auditory perception influences learned song preferences and performance of courtship behaviors in females. Taken together with previous work revealing the roles of CM and NC and the connections from those sites to their downstream targets, this emerging understanding allows us to propose a circuit model for how songs are perceived and evaluated by AIV and throughout the evaluation pathway.

### An emerging animal model of decision making

Together with the results of previous work from our group and others, this study provides additional insight into a possible circuit for sensory perception and decision making in female songbirds. A prominent feature of that circuit is the convergence of CM and NC onto AIV, and that architecture leads to questions about the cellular mechanisms through which they converge in AIV. One idea is that projections from each upstream location may converge onto one and the same cell in AIV. Alternatively, they may arrive onto different populations of cells within AIV. Defining those precise patterns of projection will be an important goal of future studies. It will also be essential to determine whether cells in AIV that receive input from those auditory areas include interneurons, projection neurons, or some combination of both. The functional consequence of this arrangement becomes apparent when we also consider the nature of the cells that AIV affects when that input arrives in VTA. Previous speculation about the connection from AIV to VTA posited that AIV projects to and activates VTA’s interneurons, inhibiting the activity of projection neurons in VTA and thus inhibiting the release of DA onto downstream targets (Kearney et al., 2019). Within the evaluation pathway, we propose a similar arrangement of inputs from CM and NC onto AIV. Specifically, we propose that CM and NC project primarily or exclusively onto interneurons within AIV (Figure 7). The net effect of this posited connectivity would be that greater activity in CM and NC projection neurons would be associated with lesser activity in AIV projection neurons, and that in turn would be associated with greater activity in VTA projection neurons (Figure 7). Thus, greater activity in CM and NC would be associated with a greater amount of DA release, and this is consistent with the observed link between greater activity in CM and NC and stronger song preferences (Elie et al., 2019; Lawley et al., 2022; MacDougall-Shackleton et al., 1998; Tomaszycki and Blaine, 2014).

A primary implication of the proposed circuit model is that activation of dopaminergic cells in VTA plays an important role in reward and motivation underlying the associated behavioral output (Figure 7). In addition to the connection from AIV to VTA, there are connections through which activity may be modulated in the dopaminergic output of this circuit (Figure 7). Projections from VP to VTA have been shown to be functionally significant, as changes in activity of neurons in VP induce changes in the activity of neurons in VTA (Chen et al., 2019; Kearney et al., 2019). Interest in the possible contributions of these three sites (AIV, VTA, and VP) becomes even greater in light of the projection from AIV to VP (Figure 7; Chen et al., 2019; Kearney et al., 2019). Thus, AIV can influence activity in VTA directly and/or indirectly through projections to VP. VP is both a receiver of output from an important stage of the evaluation pathway (AIV to VP), and it is a provider of input to a potentially more important stage of that same pathway (VP to VTA). In this context, VP becomes a very intriguing target for future study.

One idea is that AIV and VP may work together to bidirectionally encode perception of song value. For example, preferred songs may be associated with greater activity in projection neurons (PN) from CM and NC to AIV, lesser activity in AIV_PN_, and thus greater activity in VTA_PN_ and greater amounts of DA release (Figure 7). In contrast, less preferred songs may be associated with the opposite pattern of activity and lesser amounts of DA release. Thus, perceived song quality could be encoded by both increases and decreases in the amount of DA release, enabling more precise resolution of perceived value. This precision is dependent on activity in both AIV and VP. VP, however, receives inputs from sources apart from AIV, so VP likely remains quite active even when AIV is inactivated as in the experiments reported here (Chen et al., 2021; Gale et al., 2008). If this residual activity in VP were sufficient to drive activity in VTA neurons even after AIV was inactivated, we would expect that lesions of AIV would result in less precise VTA activity and consequently less stable preferences. This is in agreement with the results we observed, lending support to the ideas posited in our testable model and motivating additional experiments to further resolve the neural mechanisms through which song preference and mate choice emerge.

### Remaining questions and future directions

This study reveals that AIV is a contributing element in the song evaluation pathway. The connection between AIV and the dopaminergic center VTA offers a plausible mechanistic explanation for how activity in AIV influences evaluation. This possibility and the structures posited in our circuit model raise important questions to be addressed in future studies. For example, are the connections proposed in our model present in female Bengalese finches and other species just as they are in male zebra finches (Mandelblat-Cerf et al., 2014). Many projections in the auditory system are present in both males and females (Nottebohm and Arnold, 1976; Shaughnessy et al., 2019). Confirmation of those connections, such as those that link AIV, VP, and VTA, will be essential in studies of how activity in specific pathways is related to specific aspects of song evaluation and mate choice. Furthermore, are the effects of AIV similar in each hemisphere, or are there perhaps lateralized differences in the effect of activation or inactivation of only one side? Such precise control of individual pathways can be achieved using optogenetic approaches (reviewed in Kim et al., 2017). Optogenetic techniques could be used here to activate and deactivate AIV without disrupting activity in other areas and without accidentally activating fibers that course through AIV. By strategically placing light sources and sites of viral transfection, additional experiments could also test the functional role of specific pathways throughout this proposed circuit. The present study is a first step along the path of identifying how the song evaluation pathway functions within songbirds, specifically in understanding the process of song evaluation and how auditory signals impact decision making in receivers.

## Data Availability

The original contributions presented in the study are included in the article/supplementary material, further inquiries can be directed to the corresponding author.
